# Multikinase inhibitor sorafenib prevents pressure overload-induced left ventricular hypertrophy in rats by blocking the c-Raf/ERK1/2 signaling pathway

**DOI:** 10.1186/1749-8090-9-81

**Published:** 2014-05-09

**Authors:** Arezoo Daryadel, Anna Bogdanova, Max Gassmann, Xavier Mueller, Gregor Zünd, Burkhardt Seifert, Christine Lehalle, Nelly Frossard, Reza Tavakoli

**Affiliations:** 1 Institute of Physiology, University of Zurich, Zurich, Switzerland; 2 Institute of Veterinary Physiology, University of Zurich, Zurich, Switzerland; 3 Zurich Center for Integrative Human Physiology, University of Zurich, Zurich, Switzerland; 4 Department of Cardiac Surgery, Canton Hospital Lucerne, Lucerne, Switzerland; 5 Swiss Center for Regenerative Medicine and Clinic for Cardiovascular Surgery, University Hospital Zurich, Zurich, Switzerland; 6 Division of Biostatistics, Institute of Social and Preventive Medicine, University of Zurich, Zurich, Switzerland; 7 Laboratoire d’Innovation Thérapeutique, Unité Mixte de Recherche 7200, Centre National de la Recherche Scientifique-Université de Strasbourg, Faculté de Pharmacie, F-67400, Illkirch, France

**Keywords:** Left ventricular hypertrophy, Sorafenib, c-Raf, ERK1/2

## Abstract

**Background:**

Left ventricular hypertrophy (LVH) is a potent risk factor for sudden death and congestive heart failure.

**Methods:**

We tested the effect of sorafenib, a multikinase inhibitor (10 mg/kg, given orally, starting 2 days prior to banding, till sacrifice on day 14), on the development of LVH following aortic banding in rats.

**Results:**

The latter resulted in significant LVH caused by both an increase in cardiomyocyte volume and interstitial collagen deposition. The observed LVH was entirely blocked by sorafenib downregulating both of these components. LVH was associated with PDGF-BB and TGFβ1 overexpression, as well as phosphorylation of c-raf and ERK1/2. Additionally, the transcription factors c-myc and c-fos leading to proliferation as well as the hypertrophy-inducing transcription factor GATA4 and its regulated gene ANP were all upregulated in response to aortic banding. All these overexpressions and upregulations were inhibited upon sorafenib treatment.

**Conclusion:**

We show that sorafenib exhibits a regulatory role on the occurrence of LVH following AB in rats by blocking the rise in growth factors PDGF-BB and TGFβ1, activation of the corresponding c-Raf-ERK1/2 signaling pathway and effector mechanisms, including GATA4 and ANP. This effect of sorafenib may be of clinical importance in modulating the maladaptive hypertrophic response to pressure overload.

## Background

Human left ventricular hypertrophy (LVH) is a feature of myocardial remodeling that may occur as a consequence of compensatory mechanisms to manage increased pressure overload as observed in essential arterial hypertension or aortic valve stenosis. LVH is a potent risk factor for cardiac arrhythmias and sudden death, diastolic dysfunction and congestive heart failure [1]. Adult cardiomyocytes are unable to divide and respond to stress and growth stimuli by increasing their rate of protein synthesis, resulting in increased cell volume [2,3]. In addition to cardiomyocyte hypertrophy, pressure-overload causes interstitial cell proliferation and extracellular matrix production with increased perivascular and interstitial collagen deposition in spontaneously hypertensive rats [4]. Pressure-overload results in the local release of ligands in cardiac tissue, including growth factors such as platelet-derived growth factor (PDGF) activating its tyrosine kinase receptors [5,6], and transforming growth factor β (TGF β) activating its serine-threonine kinase receptors [7-9]. The signaling pathways include the cascade of c-Raf (serine-threonine kinases) and its downstream substrates mitogen-activated protein kinases (MAPK) [5,10,11]. The extracellular signal-regulated kinases (ERK)1/2, a subgroup of MAPK, play a critical role in the regulation of gene expression leading to cardiac hypertrophy [12]. Activation of ERK1/2 by phosphorylation has been associated with upregulation of cardiac transcription factors c-myc, c-fos and GATA4 [5,10]. GATA4 is a critical transcription factor of most cardiac-expressed structural genes and hypertrophy-related genes, including the atrial natriuretic peptide (ANP) gene [13,14]. In humans, mutations in c-Raf are associated with cardiac hypertrophy in genetic disorders such as Noonan syndrome suggesting an important role of c-Raf in pathophysiological modulation of cardiac hypertrophy [15].

Data accumulated from cancer research indicate a variety of theoretical parallels between signaling pathways that drive tumorigenesis [16,17], and those that regulate cardiac hypertrophy [5,10,11], a major common signal transduction pathway being represented by the Raf-ERK1/2 cascade. Sorafenib is a multiprotein kinase inhibitor used as an antineoplasic agent, inhibiting the Raf serine/threonine kinase, as well as the VEGFR and PDGFR-β tyrosine kinases, and the c-kit tyrosine kinase. Recent studies show that sorafenib prevents the progression of right ventricular hypertrophy due to pulmonary hypertension in a rodent experimental model [18,19]. This prompted us to evaluate the effect of sorafenib on the development of left ventricular hypertrophy in a rodent experimental model of mechanically-induced pressure overload obtained by banding of the supra-renal aorta.

## Methods

### Experimental design

#### Animals

Male Lewis rats (Harlan, Holland) weighing 90-110 g were used and received humane care in strict accordance with the recommendations in the Guide for the Care and Use of Laboratory Animals of the National Institutes of Health. The protocol was approved by the Committee on the ethics of Animal Experiments of the Canton Zurich, Switzerland (Permit Number 122/2008). All surgery was performed under sodium pentobarbital anesthesia, and all efforts were made to minimize suffering. Animals were maintained in standard housing conditions with dry diet and water available *ad libitum*.

#### Treatment groups

Animals were randomly assigned to receive sorafenib 10 mg/kg (Bayer, Germany) or vehicle (NaCl 0.9%) once daily by gavage, starting 2 days prior to surgery and continuing for 14 days thereafter. They were killed 12–14 hours after the last gavage, and blood and tissue harvested for subsequent analyses.

#### Surgery

Sorafenib- or solvent-treated animals were further randomly assigned to undergo aortic banding (AB) or sham operation. In banding animals, a median laparotomy was performed, the supra-renal aorta dissected free, and a ring of polyurethane (1 mm diameter) placed around the aorta and secured with 7/0 Prolene as described [20]. In control sham animals, a median laparotomy was performed and the supra-renal aorta dissected free without any placement of a ring. After euthanasia, the left myocardial ventricle was rapidly excised and weighed before tissue processing. The ventricular weight to body weight (VW/BW) ratio was used as the macroscopic indicator of left ventricular hypertrophy.

### Histological analysis

After excision, the left myocardial ventricle was rapidly fixed in 10% formalin, and paraffin-embedded. Sections (7-μm) were processed for hematoxylin and eosin (H & E), and van Gieson’s staining (Elastica van Gieson Kit, Merck, Darmstadt, Germany) to detect collagen deposition (red color) indicative of cardiac fibrosis [21]. The stained sections were visualized under light microscopy (Vanox-S, Olympus, Tokyo, Japan), and photographed with a digital camera (U-PMTVC, Olympus, Tokyo, Japan). The cross-sectional area of cardiomyocytes was measured using the ImageJ 1.30v Software system in at least 10 different areas for each section. Results were obtained from 2–4 sections per rat for a total of 5 rats selected at random from the various treatment groups.

### PDGF-BB and TGF-β1 ELISA

The ventricular levels of PDGF-BB and TGF-β1 were determined by using commercial ELISA kits (R & D Systems, Minneapolis, MN) according to the manufacturer’s recommendations. Briefly, 100 mg of snap-frozen ventricle tissue was homogenized in 500 μl of lysis buffer containing 50 mM Tris HCl [pH 7.5], 150 mM NaCl, 10 mM EDTA, 0.25% Triton X-100, 0.1% NP-40, 1 mM PMSF and cocktail protease inhibitors. The lysates were clarified at 14,000 *g* for 15 min at 4°C. ELISA levels were normalized to the total protein level (BCA Protein Assay kit, Pierce, Rockford, USA) for each sample. All measurements were performed in duplicate.

### Reverse transcription and quantitative polymerase chain reaction (RT-qPCR)

Total RNA was extracted from the ventricular myocardium using RN*easy*® Mini kit, including DNase treatment (Qiagen, Basel, Switzerland). Total RNA (2.5 μg) was reverse transcribed using a High-Capacity cDNA Archive Kit (Applied Biosystems, Rotkreuz, Switzerland) and MultiScribe reverse transcriptase (50 units/μl), random hexamers, dNTPs, and RNase inhibitor (20 units/μl) (all from Applied Biosystems). The reaction was performed in a total volume of 25 μl in a GeneAmp PCR thermocycler (Applied Biosystems) at 25°C for 10 min, followed by 2 hours at 37°C, and 10 min at 70°C. Samples without enzyme in the RT reaction were used as negative controls to evidence the absence of contamination with genomic DNA. Predeveloped primer probes were used for ANP (Rn00561661_m1), GATA4 (Rn01530459_m1), c-myc (Rn00561507_m1) and c-fos (Rn02396759_m1) as well as the reference genes 18S rRNA and GAPDH (all from Applied Biosystems). Real-time quantitative PCR was performed on an ABI PRISM 7500 Sequence Detection System (“TaqMan”, Applied Biosystems, Darmstadt, Germany) using heat-activated TaqDNA polymerase (Amplitaq Gold; Applied Biosystems). After an initial 2 min at 50°C, and 10 min at 95°C, the samples were submitted to 40 cycles comprising 15 sec at 95°C for denaturation and 60 sec at 60°C for hybridization and elongation. The expression of candidate genes was normalized to two reference genes (18S rRNA and GAPDH) giving comparable results and analyzed by the delta Ct method (Applied Biosystems).

### Collagen measurement

Total soluble collagen in whole ventricle homogenate was quantified using the SirCol collagen assay (Biocolor, Belfast, UK). The ventricle homogenate of vehicle- or sorafenib-treated sham and aortic banding animals were prepared at day 14. Sirius Red (1 ml), an anionic dye that reacts specifically with (Gly-X-Y)_n_ tripeptide in the triple-helix sequence of mammalian collagens under assay conditions, was added to ventricle homogenate (10 μl), and incubated under gentle rotation for 30 min at room temperature. After centrifugation for 10 min at 12,000 *g*, the pelleted collagen-bound dye was dissolved with 1 ml of 0.5 M NaOH, and the absorbance was read at 540 nm. Results are presented as a ratio to the total protein content. All measurements were performed in duplicate.

### Western blot analysis

The ventricle lysate was prepared in 50 mM Tris HCl [pH 7.5], 150 mM NaCl, 10 mM EDTA, 0.25% Triton X-100, 0.1% NP-40, 1 mM PMSF and cocktail inhibitors (Roche). Protein concentration was measured by the BCA technique. Proteins were separated (100 μg per lane) on an SDS-polyacrylamide gel and transferred onto nitrocellulose membranes. Membranes were blocked for 1 hour at room temperature in 5% non-fat milk with 0.1% Tween 20 in Tris Buffer Saline (pH 7.4), and were probed overnight at 4°C with antibodies: rabbit anti-rat phospho-ERK1/2 (Thr202/Tyr204), mouse anti-rat total ERK1/2 (R & D biosystems; 1:1000), rabbit anti-rat phospho-c-Raf (Ser259) and rabbit anti-rat total c-Raf (Cell signaling; 1:1000). Blots were washed in TBS/0.1%-Tween-20 for 1 hour at room temperature, and incubated with the appropriate HRP-conjugated secondary antibody (Jackson ImmunoResearch Laboratories; 1/2000) in TBS/0.1% Tween-20/5% nonfat milk for 1 h. Bound antibodies were visualized using enhanced chemiluminescence ECL kit (Perkin-Elmer, Boston, MA) according to the manufacturer’s instructions. Evaluation of the expression of specific proteins was performed with the ImageJ 1.30v Software system.

### Statistical analysis

All data are results of 6 to 8 *in vivo* experiments, and are presented in figures as means ± SD (bars). An analysis of variance (ANOVA) followed by Tukey’s multiple comparisons test was performed to analyze the modulating effect of sorafenib on the development of LVH following aortic banding. Statistical analyses were performed using PRISM 6 GraphPad software (GraphPad, La Jolla, CA, USA). P-values <0.05 were considered significant.

## Results

### Left ventricular hypertrophy

**Figure 1:**
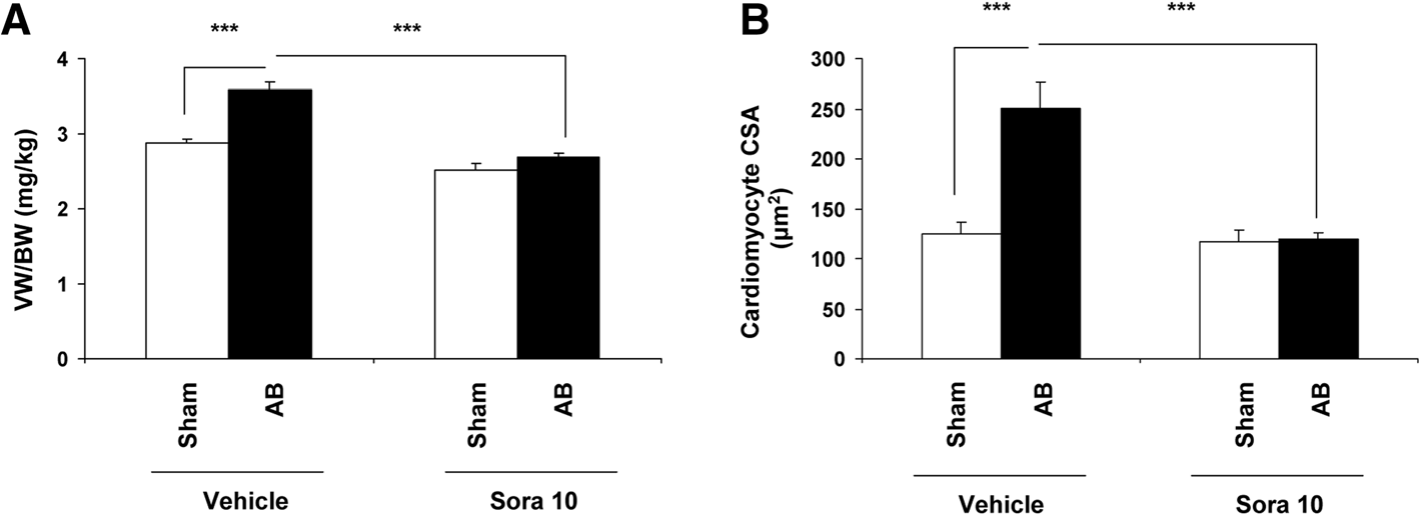
**Sorafenib prevents the development of pressure overload**-**induced left ventricular hypertrophy** (**LVH**) **and cardiomyocyte volume. A**. Following aortic banding (AB), LVH is anatomically evidenced by a significant rise in the ventricular weight to body weight (VW/BW) ratio (p < 0001, ***), which was blocked by sorafenib (p < 0001, ***). **B**. The observed LVH following AB is histologically accounted for by an increase in cardiomyocyte volume reflected by a significant increase in the cardiomyocyte cross-sectional area (CSA) expressed in μm2 (p < 0001, ***). This component of LVH was totally blocked by sorafenib treatment (p < 0.0001, ***). Cardiomyocyte CSA was measured in 10 different areas per section. Results were obtained from 2–4 sections per rat for a total of 6 rats selected at random from each group.

Aortic banding in vehicle-treated animals induced significant left ventricular hypertrophy evidenced by a 25% increase in the VW/BW ratio (sham vehicle 2.9 ± 0.1 *vs* aortic banding vehicle 3.6 ± 0.3 mg/kg, p < 0.0001). This hypertrophic response significantly decreased after sorafenib treatment (sham sorafenib 2.5 ± 0.2 *vs* aortic banding sorafenib 2.7 ± 0.2, p < 0.2, and p < 0.0001 between banding vehicle and banding sorafenib) (Figure 1A).

**Figure 2:**
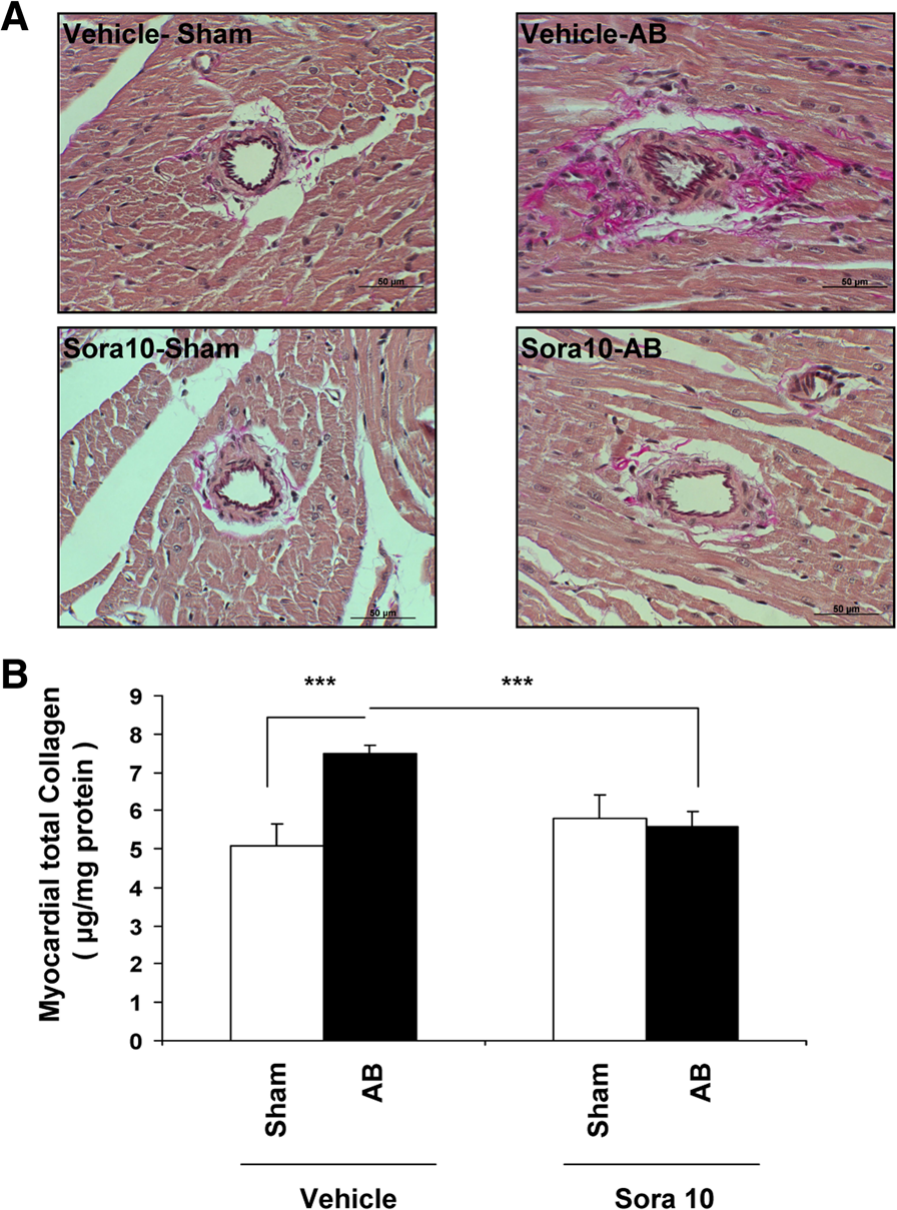
**Sorafenib blocks interstitial collagen deposition following aortic banding. A**. Interstitial collagen deposition following AB is evidenced by van Gieson’s staining and predominantly localizes to perivascular spaces. Sorafenib prevents interstitial collagen deposition after AB. **B**. Quantitative measurement of tissue collagen by SirCol assay normalized to total protein content (μg/mg). Sorafenib blocks this component of LVH (p < 0001, ***). All measurements were performed in duplicate.

Aortic banding resulted in an increase in left ventricular cardiomyocyte cross-sectional area (sham vehicle 124.2 ± 28.0 *vs* aortic banding vehicle 250.8 ± 78.1 μm^2^, p < 0.0001) (Figure 1B), as well as interstitial matrix deposition quantified by myocardial total collagen (sham vehicle 5.1 ± 1.2 *vs* aortic banding vehicle 7.5 ± 0.4 μg/mg protein, p < 0.0001) (Figure 2A and 2B) (p < 0.0001, Figure 2B). Sorafenib entirely prevented left ventricular hypertrophy by abolishing both cardiomyocyte hypertrophy (sham sorafenib 117.2 ± 19.8 *vs* aortic banding sorafenib 119.7 ± 10.9 μm^2^, p = 0.2) and interstitial matrix deposition (sham sorafenib 5.8 ± 1.1 *vs* aortic banding sorafenib 5.6 ± 0.8 μg/mg protein, p = 0.2, and p < 0.0001 between banding vehicle and banding sorafenib) (Figure 2A and 2B).

### Growth factor expression

**Figure 3:**
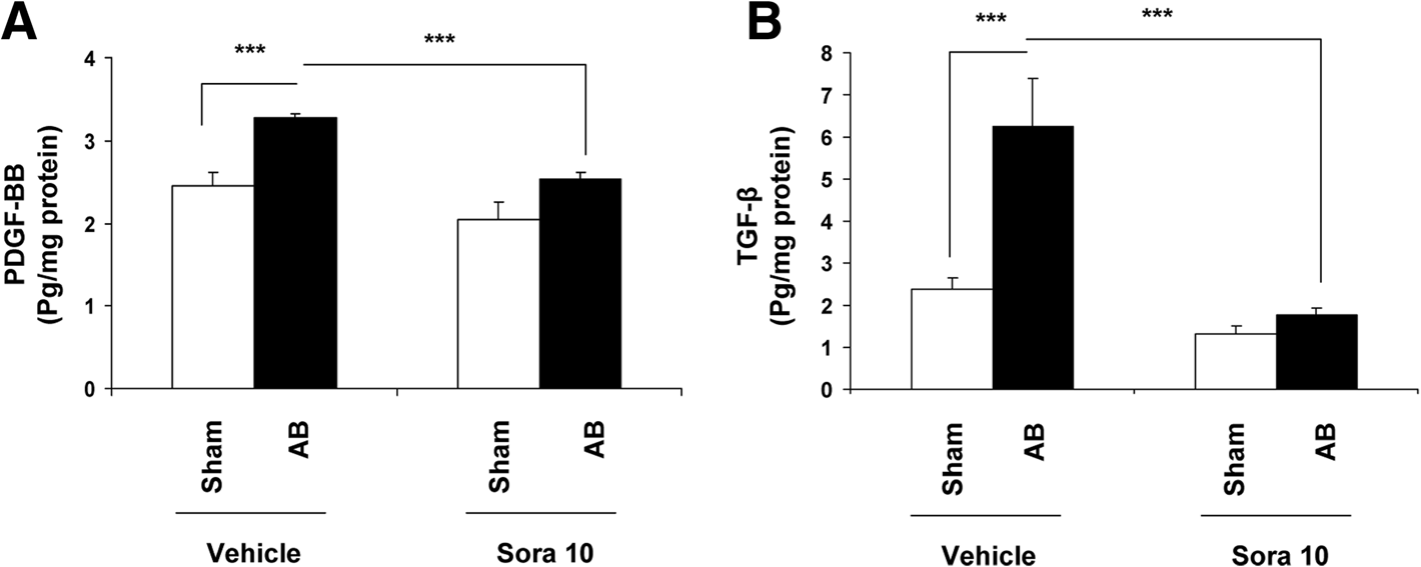
**Sorafenib inhibits the over expression of growth factors in myocardium.** Tissue levels are determined by ELISA normalized to total protein content (pg/mg). **A**. Sorafenib abolishes the over expression of myocardial PDGF-BB levels in banded animals (p < 0001, ***). **B**. Sorafenib suppresses over expression of the pro-fibrotic growth factor TGFβ1 in myocardium in banded animals (p < 0001, ***). All measurements were performed in duplicate.

Pressure overload-induced left ventricular hypertrophy was associated in vehicle-treated animals with significant increase in myocardial content of the pro-hypertrophic factors platelet-derived growth factor BB (PDGF-BB) (1.3-fold increase, Figure 3A) and transforming growth factor β1 (TGF- β1) (2.5-fold, Figure 3B) (p < 0.0001 for banding vehicle *vs* sham vehicle for both). Sorafenib treatment totally abolished the overexpression of both myocardial growth factors PDGF-BB (Figure 3A) and TGF-ß1 (Figure 3B) in banding animals (p < 0.0001 for vehicle *vs* sorafenib).

### Signaling pathways

**Figure 4:**
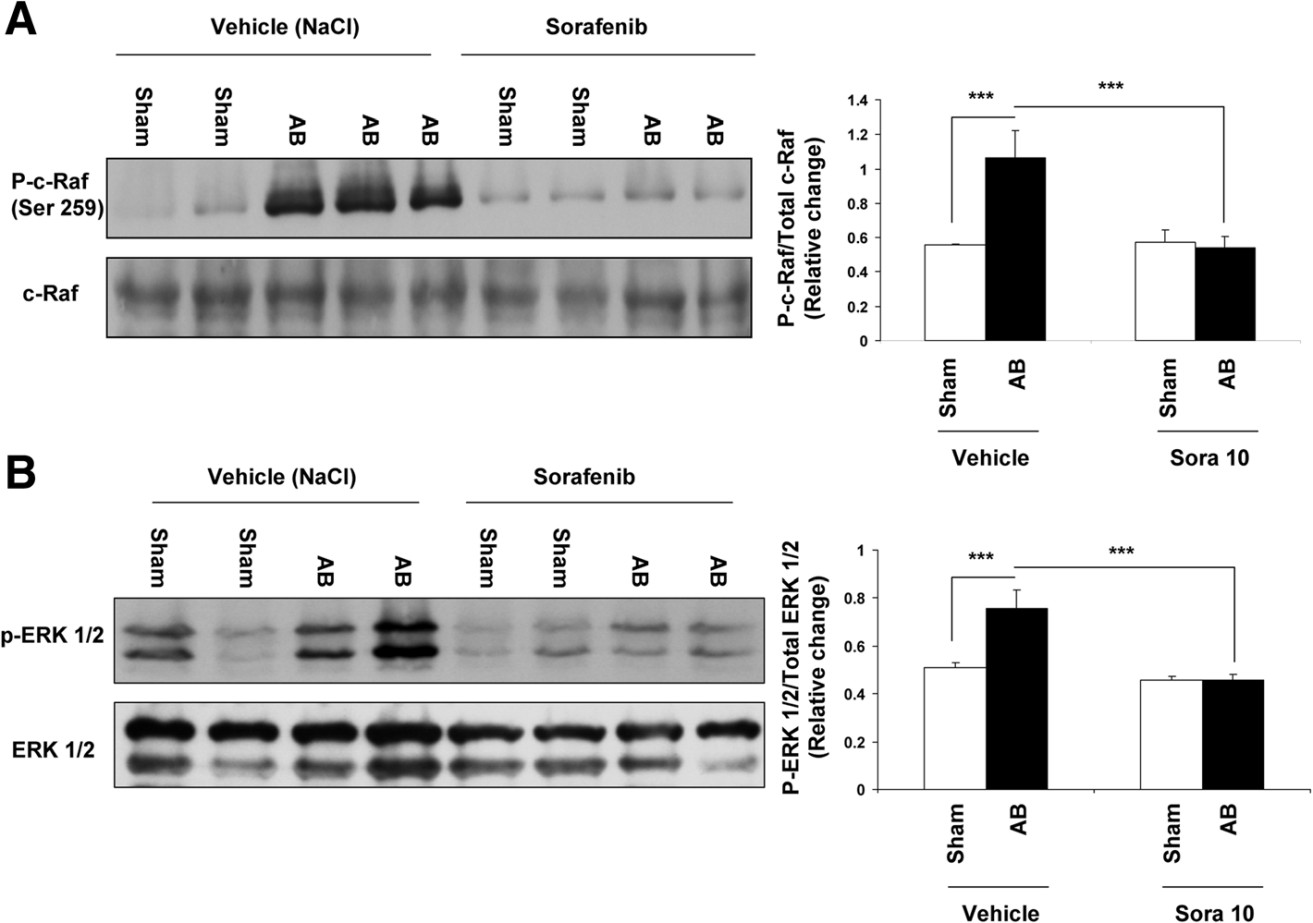
**Sorafenib prevents phosphorylation of c**-**Raf and ERK1**/**2 in myocardium following AB. **Left panel: representative Western blot for phosphorylated (upper band) and total (lower band) kinases from ventricle tissue. Right panel: ratios of phosphorylated to total kinases are presented as histograms. **A**. c-Raf phosphorylation is inhibited by sorafenib treatment in banded animals (p < 0001, ***). **B**. ERK1/2 phosphorylation is blocked by sorafenib treatment in banded animals (p < 0001, ***).

Aortic banding resulted in a 2.2-fold increased phosphorylation of c-Raf (Figure 4A) and of its downstream kinase ERK1/2 (1.8-fold increase, Figure 4B) (p < 0.0001 for banding vehicle *vs* sham vehicle for both). Sorafenib treatment totally abolished the increased phosphorylation of c-Raf as well as ERK 1/2 (p < 0.0001 for banding vehicle *vs* banding sorafenib for both).

### Expression of the pro-proliferative and pro-hypertrophic transcription factors

**Figure 5:**
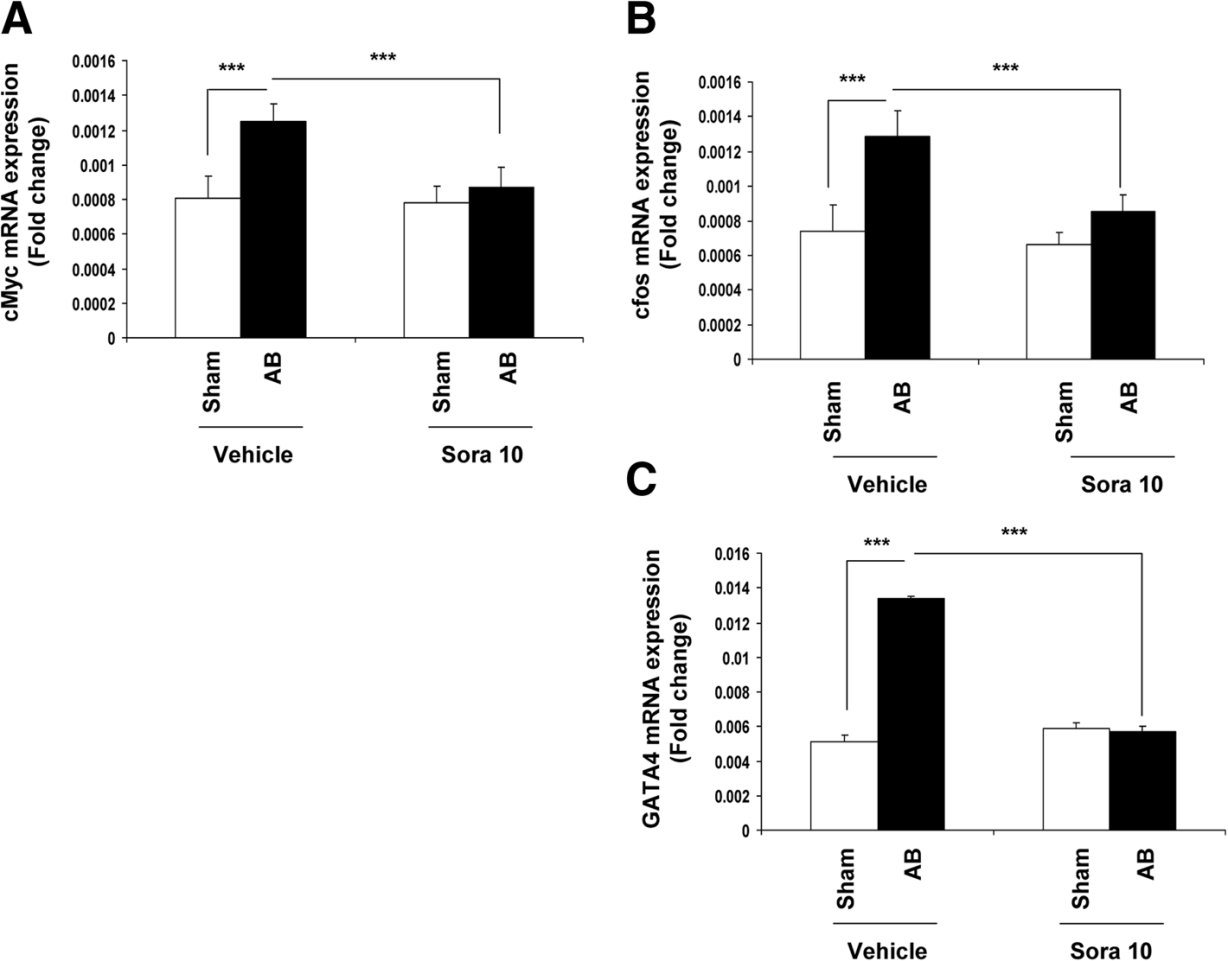
**Sorafenib regulates the increased expression of nuclear transcription factors resulting from AB.** mRNA of c-myc **(A)** and c-fos **(B)** and GATA4 **(C)** are measured by reverse transcription quantitative polymerase chain reaction (RT-qPCR), and normalized to the reference gene GAPDH. Increased expression of pro-proliferative transcription factors c-myc **(A)** and c-fos **(B)** and pro-hypertrophic factor GATA4 **(C)** in AB as compared to sham-operated animals (p < 0.0001, *** for banding vehicle versus sham vehicle for all three factors) is inhibited by sorafenib (p < 0.0001, *** for banding vehicle versus banding sorafenib for all three factors). All measurements were performed in duplicate.

Activation of the c-Raf/ERK pathway following AB was accompanied in vehicle-treated animals by a significant increase in the expression of pro-proliferative nuclear transcription factor mRNA: 1.7-fold increase in c-myc (Figure 5A) and 2.1-fold increase in c-fos (Figure 5B). Additionally, a 2.6-fold increase in the expression of the pro-hypertrophic transcription factor GATA4 was observed (Figure 5C) (p < 0.0001 for banding vehicle *vs* sham vehicle for all three factors).

Sorafenib totally abolished the increase in nuclear transcription factor mRNA expression, c-myc (Figure 5A), c-fos (Figure 5B) and GATA4 (Figure 5C) in banding animals (p < 0.0001).

### Expression of the pro-hypertrophic factor ANP mRNA

**Figure 6:**
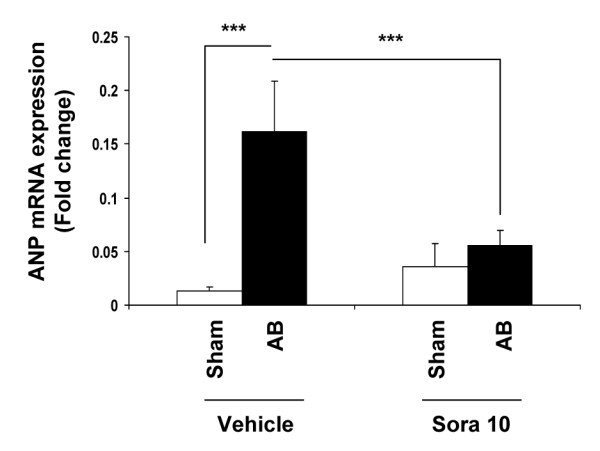
**Sorafenib abolishes up**-**regulation of the pro**-hypertrophic factor gene atrial natriuretic peptide ANP in banded animals (p < 0001, ***). ANP mRNA is measured by RT-qPCR and normalized to the reference gene GAPDH. All measurements were performed in duplicate.

Transcription of the pro-hypertrophic factor ANP is under direct control of GATA4. As expected, ANP mRNA expression was enhanced in vehicle-treated AB *vs* sham-operated animals, and reached a 10-fold increase after aortic banding (p < 0.0001, Figure 6). Again sorafenib totally abolished the increase in ANP mRNA expression in banding animals (p < 0.0001, Figure 6).

## Discussion

**Figure 7:**
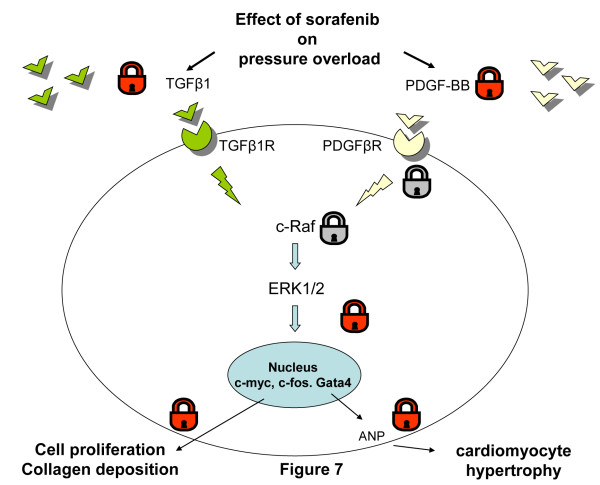
**Schematic representation of the blocking effect of sorafenib on development of left ventricle hypertrophy** (**LVH**) **induced by aortic banding.** Mechanical stretch induced by pressure overload results in over-expression of growth factors, PDGF-BB and TGFβ1 in myocardium [5]. The original finding of this study shows sorafenib to inhibit the up-regulation of these growth factors following AB. Phosphorylation and hence activation of PDGFβR and TGFβ1R is known to activate the downstream signaling pathway of c-Raf/ERK1/2 [10]. Sorafenib is reported to block phosphorylation and activation of PDGFβR and c-Raf in cancer studies [23]. By blocking the c-Raf/ERK1/2 cascade, sorafenib down-regulates the expression of pro-proliferative (c-myc, c-fos) and pro-hypertrophic (GATA4) transcription factors as well. One of the pro-hypertrophic effector mechanisms regulated by GATA4 is ANP [14] which is in turn suppressed by sorafenib treatment. The sum of the inhibitory effects of sorafenib ends at the suppression of cardiomyocyte hypertrophy as well as of interstitial collagen deposition, the two main components of LVH. Blocking effect of sorafenib on expression of growth factor as demonstrated in this study. Blocking effect of sorafenib previously reported by Wilhelm et al. [23].

Our study reports that the pressure overload induced by banding of the supra-renal aorta in Lewis rats resulted in significant LVH that was entirely blocked by treatment with the multikinase inhibitor sorafenib. This mechanically-induced pressure overload following banding closely mimics the clinical setting of patients with left ventricular hypertrophy due to aortic valve stenosis or essential arterial hypertension [3,20]. This left ventricular hypertrophy was associated with PDGF-BB and TGFß1 overexpression, as well as with phosphorylation of their c-raf and ERK1/2 signaling pathways, all of which were downregulated by sorafenib treatment. The transcription factors c-myc and c-fos leading to proliferation were also upregulated in response to aortic banding, as well as the hypertrophy-inducing transcription factor GATA4 and its regulated gene ANP. All of these were downregulated by sorafenib treatment as resumed in Figure 7.

Sorafenib was administered as a daily oral treatment at the dose of 10 mg/kg, as based on experimental reports in the rat [18,19,22], in preclinical murine models of cancer [23,24], as well as in cancer patients [24]. Sorafenib is reported to inhibit in particular the Raf serine/threonine kinase isoforms both *in vitro* and *in vivo* in various cancer models in the mouse and rat [24]. This signaling pathway is described as playing a central regulatory role in the genesis of pressure overload-induced hypertrophy [5,10]. In patients with Noonan and Leopard syndromes, presenting genetic disorders involving mutations in c-Raf, cardiac hypertrophy is one of developmental organ anomalies suggesting an important role of c-Raf in pathophysiological modulation of cardiac hypertrophy [15]. In accordance with this, we show here that pressure overload-induced left ventricular hypertrophy leads to the Raf cascade activation as evidenced by raf and ERK phosphorylation, and that this activation was blocked by sorafenib treatment. In addition, sorafenib is reported to inhibit the PDGFRß tyrosine kinase [25]. Our study adds here the new information that sorafenib decreases production of the PDGFRß agonist platelet-derived growth factor BB (PDGF-BB) whose expression is increased in animals presenting LVH following aortic banding. PDGF-BB is a growth factor that may in this study account for the increase in cardiomyocyte volume evidenced on histological cross-sections. Such a role was also reported in the development of myocardial hypertrophy in spontaneously hypertensive rats [26]. Thus, both inhibition of c-Raf and reduction in growth factor production may have participated in prevention of ERK 1/2 phosphorylation and activation, and thereby in prevention of left ventricular hypertrophy by sorafenib.

On the other hand, we show here a rise in the pro-fibrotic TGF-β1 in cardiac tissue after banding, and this was associated with enhanced interstitial collagen deposition, as evidenced by collagen measurements and van Gieson’s localization. Sorafenib inhibited both the rise in TGFβ1 and collagen in cardiac tissue, thus preventing the essential component of left ventricular hypertrophy, i.e. interstitial collagen deposition.

In line with other reports [5,10], the phosphorylation and activation of c-Raf-ERK1/2 signaling pathway was accompanied with up-regulation of the cardiac transcription factors c-myc and c-fos implicated in cell proliferation and fibrosis. As expected, sorafenib down-regulated the expression of these transcription factors, resulting from blockade of the ERK1/2 pathway by sorafenib as reviewed in Gupta *et a*l. [3]. Indeed, phosphorylation of ERK1/2 induces increases in both c-myc [12] and c-fos expression [27], thereby allowing interstitial cell proliferation, fibrosis and collagen deposition, all of which being blocked by sorafenib.

Activation of the c-Raf-ERK1/2 pathway also activates the cardiac transcription factor GATA4 in cardiomyocyte hypertrophy [5], which is in accordance with results reported here. A large body of evidence suggests that GATA4 is an important regulator of cardiac genes involved in pressure overload-induced cardiac hypertrophy [5,13], including the hypertrophic agent ANP [14,28]. Indeed, we show an increased expression of GATA4 and of ANP, the latter reaching 10-fold increase following pressure overload induction in this study. In this line of evidence, our report clearly shows that sorafenib suppressed expression of both the pro-hypertrophic effector ANP and its transcription factor GATA4, and that sorafenib may therefore be an interesting treatment strategy for cardiac hypertrophy.

The present study evidences a preventive effect of sorafenib, that was administered prior to induction of mechanical pressure overload during a relatively short period of 2 weeks. The choice of this observation period was based on the fact that in this model left ventricular hypertrophy was already present at one week and near maximal at two weeks following aortic banding (personal data). The effect of sorafenib on established left ventricular hypertrophy, and of its administration over a longer period in healthy and in (de)-compensated hypertrophic hearts will need to be further addressed, together with modifications of hemodynamic parameters that could account for the survival of banded animals under sorafenib.

Recent evidence indicates that in some instances, cardiotoxicity occurs after use of kinase inhibitors including sorafenib in cancer patients [29]. With regard to sorafenib, acute coronary syndrome and arterial hypertension have been reported in a limited number of patients treated for renal cell carcinoma or melanoma [30]. The mechanisms involved in the cardiotoxicity of sorafenib remain presumptive. While inhibition of c-Raf by sorafenib has been sggested as underlying its cardiotoxicity [29], it was also submitted that sorafenib did not exert its damaging effects through RAF inhibition of the RAF/MEK/ERK kinase cascade [31]. Our finding of inhibition of phosphorylation and activation of ERK1/2 by sorafenib after aortic banding is in clear contrast with results observed *in vitro* by Hasinoff and Patel after treatment of isolated cardiomyocytes by sorafenib [31]. Their study reports an increase of the basal phosphorylation of ERK1/2 by sorafenib *in vitro*. Different experimental models, as unstimulated *in vitro* (31) *and* in pathological situation *in vivo* (our study) may account for these opposed effects of sorafenib on c-Raf/ERK pathway.

## Conclusions

We show here that sorafenib exhibits a regulatory role on the occurrence of left ventricular hypertrophy by blocking the rise in growth factors, and activation of the corresponding c-Raf-ERK1/2 signaling pathway and effector mechanisms including ANP production. This effect of sorafenib may be of importance in modulating the maladaptive hypertrophic response to pressure overload. Left ventricular hypertrophy due to systemic arterial hypertension or aortic valve stenosis is a frequently encountered situation in the clinical setting. The main new finding of this study of prevention of left ventricular hypertrophy by sorafenib in banded animals could therefore be potentially of clinical interest.

## Competing interests

The authors declare that they have no competing interests.

## Authors’ contributions

Conceived and designed the experiments: RT, AD, AB. Performed the experiments: RT, AD. Analyzed and discussed the data: RT, NF, CL, BS, MG. Contributed reagents/materials/analysis tools: GZ, XM. Wrote the paper: RT, NF. All authors read and approved the final manuscript.
